# Long-Term Follow-up of a Wide-Diameter Bone-Anchored Hearing Implant: 10-Year Experience on Stability, Survival, and Tolerability of an Implant-Abutment Combination

**DOI:** 10.1097/MAO.0000000000003763

**Published:** 2022-11-23

**Authors:** Emma Margaretha Teunissen, Coosje Jacoba Isabella Caspers, Maarten Adriaan Vijverberg, Emmanuel Antonius Maria Mylanus, Myrthe Karianne Sophie Hol

**Affiliations:** ∗ Department of Otorhinolaryngology, Donders Center for Neuroscience, Radboud university medical center, Nijmegen, the Netherlands; † Department of Otorhinolaryngology/Head and Neck Surgery, University Medical Center Groningen, University of Groningen, Groningen, the Netherlands; ‡ Research School of Behavioral and Cognitive Neurosciences, Graduate School of Medical Sciences, University of Groningen, Groningen, the Netherlands

**Keywords:** BAHI, Bone-anchored hearing implant, Early loading, Hearing loss, Holgers score, Implant stability, Implant survival, ISQ, Soft tissue reaction, Wide-diameter implant

## Abstract

To compare stability, survival, and soft tissue reactions between a wide-diameter (test) and previous-generation small-diameter (control) bone-anchored hearing implant and to ascertain the safety of loading the test implant 3 weeks after surgery, at a long-term follow-up of 10 years.

**Study Design:**

This study is a continuation of two previously completed, multicenter, randomized, controlled trials and consisted of one to two additional follow-up visits until 10 years after surgery.

**Patients:**

Fifty-one of the 72 participants from the previous trials were included. Patients received a test or control implant. All control implants were loaded 6 weeks after surgery (group A). Test implants were loaded 3 (group B) or 6 weeks (group C) after surgery.

**Results:**

The test implant showed significantly higher implant stability quotient (ISQ) values than the control implant throughout the 10-year follow-up. At 10 years, the mean ISQ-high values for both implants were higher than at the first follow-up visit. No significant differences in change of ISQ-high from baseline to 10 years were noticed between both implants and loading groups. Soft tissue reactions were rarely seen. At 10-year follow-up, no patients presented with *adverse* soft tissue reactions. Excluding explantations, the implant survival rate was 78.6% (group A), 100% (group B), and 90.0% (group C).

**Conclusions:**

The test implant showed superior mean ISQ values and significantly better implant survival throughout 10-year follow-up. In addition, the current study concludes that it is safe to load the test implant at 3 weeks after surgery, as long-term results show high ISQ values and good implant survival.

## INTRODUCTION

Hearing rehabilitation through direct bone conduction is a well-established method to overcome bilateral conductive and mixed hearing loss, as well as unilateral conductive and mixed hearing loss and single-sided deafness (1). Since its introduction in 1977 (2), the bone-anchored hearing implant (BAHI) underwent various improvements. The original auditory osseointegrated implant was a titanium implant with an as-machined surface, designed by Brånemark (3). Later, it became commercially available as the Baha flange fixture. In 2009, Cochlear introduced the BIA300 implant and abutment. The new implant was fitted with a wider-diameter fixture to increase implant stability (4) and with small-sized threads at the neck and a moderately rough surface to increase bone response (i.e., remodeling) after surgery (5). The abutment design was also renewed: the rounded shape and conical connection to the fixture, providing a tighter seal, would reduce soft tissue reactions.

Previously, Dun et al. (6) and Nelissen et al. (7) reported the 6-month and 3-year results from a multicenter, randomized controlled trial of this wide-diameter implant. Subsequently, den Besten et al. (8) reported the 5-year results. All three articles showed the superiority of this implant compared with the previous small-diameter implant in terms of implant stability and soft tissue reaction. In addition, both implants showed equally high implant survival. Based on these favorable outcomes, it was thought that the time between implantation and sound processor loading could be shortened. In clinical practice, the loading time was already reduced to 3 weeks after surgery and reported safe in a retrospective survey of more than 1,000 previous-generation BAHIs (9). Subsequently, it was hypothesized that earlier loading would also be safe for wide-diameter implants. A prospective controlled trial on loading the wide-diameter implant 3 weeks after surgery was performed to investigate this hypothesis (10,11). The outcomes of this “early loading” group were compared with the results of patients implanted with the same implant, in which the implant was loaded at 6 weeks after surgery (6,7). At 3-year follow-up, the authors concluded that loading the wide-diameter implant 3 weeks after surgery was safe in adult patients with normal bone quality. Previous research even suggested that wide-diameter implants can be safely loaded 2 weeks after surgery (12), 1 week after surgery (13,14), and even extremer: 1 day after surgery (15). However, these last-mentioned studies only had a maximum follow-up of 1 year and therefore did not provide sufficient long-term data on reduced loading times in wide-diameter implants.

This study compares stability, survival, and soft tissue reactions for the wide-diameter implant with abutment (test) and the previous-generation small-diameter BAHI (control) and aims to ascertain the safety of loading 3 weeks after surgery at a long-term follow-up of 10 years. Thereby, this study is the first to report such extreme long-term results of this wide-diameter implant-abutment combination.

## MATERIALS AND METHODS

### Study Design and Participants

This study is a continuation of two previously completed, multicenter randomized controlled trials on a wide-diameter implant's stability, survival, and tolerability. In the previous trial 1, the clinical outcomes of the wide-diameter implant (test) compared with the previous-generation implant (control) loaded 6 weeks after surgery were evaluated at 6 months (6), 3 years (7), and 5 years (8) after implantation. In trial 2, the outcomes of the wide-diameter implant loaded 3 weeks compared with 6 weeks after surgery were evaluated at 6 months (10) and 3 years (11) after implantation.

Patients were divided into three groups to simplify the interpretation of the following sections: group A, patients who received a control implant that was loaded 6 weeks after surgery; group B, patients who received a test implant that was loaded 6 weeks after surgery; and group C, patients who received a test implant that was loaded 3 weeks after surgery. Groups A and B were compared in the aforementioned trial 1, and groups B and C were compared in trial 2.

The current study consisted of one follow-up visit at 10 years after implantation for groups A and B and two additional follow-up visits for group C: at 5 and 10 years after implantation. All follow-up visits were performed at the Radboud university medical center, Nijmegen, the Netherlands. Therefore, only the Dutch patients who completed the original trials (n = 72) were approached for participation in the current study. To be included in the original trials, patients had to have a BAHI indication and be at least 18 years old at the time of implantation (6,10). Exclusion criteria were as follows: inability to follow investigational procedures, any factor at the investigator's discretion that was considered to contraindicate participation, and any disease or treatment known to compromise the bone quality at the implant site (e.g., radiotherapy, bone diseases such as osteoporosis). For patients who had lost or underwent explantation of the implant, time to implant loss was recorded. Patients who could not physically attend the 5- or 10-year follow-up visit for other reasons were included in the implant survival analysis. The last available information regarding implant survival was obtained telephonically from the patient, medical records, or data captured from the original investigations.

Two BAHI surgeons (EM and MH) performed all operations in our tertiary referral center between April 2009 and July 2010. In all cases, the surgery included a single-stage procedure using the Nijmegen linear incision technique with tissue reduction (16). Sound processor loading was performed as of 6 weeks after surgery for groups A and B (mean, 6 weeks and 0 days) and within a 3-week ± 1-day visit window (mean, 3 weeks and 1.5 days) after surgery for group C. Loading could only proceed if the implant's stability and soft tissue status were subjectively judged to be sufficient based on clinical assessment by the investigator.

All trials followed a similar follow-up scheme. In trial 1, the follow-up visits took place at 10 days, 4 weeks, 6 weeks, 8 weeks, 12 weeks, 6 months, 12 months, 24 months, 36 months, 5 years, and 10 years after implantation. Follow-up visits in trial 2 took place at 10 days, 3 weeks, 6 weeks, 12 weeks, 6 months, 12 months, 24 months, 36 months, 5 years, and 10 years after implantation.

### Implants

The test implant, used in groups B and C, included the wide-diameter titanium implant (diameter, 4.5 mm; length, 4 mm) with 6-mm rounded, apically converging titanium abutment developed by Cochlear Bone Anchored Solutions AB (Mölnlyncke, Sweden). With an additional minor change to the internal abutment connection design, this system was later commercialized under the name Cochlear Baha BIA300 Implant and Abutment. The control implant, used in group A, included the previous-generation as-machined titanium Cochlear Baha flange fixture (diameter, 3.75 mm; length, 4 mm) with 6-mm conically shaped abutment from the same manufacturer. In addition to the difference in abutment shape and the wider diameter, the test implant incorporates small-sized threads at the implant neck and the moderately rough TiOblast (Dentsply, Mölndal, Sweden) surface on the intraosseous part of the implant (6–8).

### Outcome Measures

For all patients who attended the 5- and/or 10-year follow-up visit(s), relevant medical history since the last study visit, implant stability, soft tissue reactions, implant loss or explantation, and sound processor usage were recorded.

Implant stability was objectively measured with resonance frequency analysis (RFA) and subsequently expressed as an implant stability quotient (ISQ) (17–19) value. Using RFA involves sending and reading magnetic pulses via a handheld Osstell ISQ device (Ostell AB, Göteborg, Sweden) to a SmartPeg (type 43 for groups A and B and type 55 for group C from 12 weeks onward) attached to the abutment. Perpendicular measurements with the handheld device result in two values recorded as an ISQ-high value and an ISQ-low value. ISQ values can range from 0 to 100, with increasing scores presenting a more rigid bone-implant interface. Because the ISQ score also represents other implant variables such as abutment length and skin thickness, assessing changes over time is more sensible than evaluating absolute values at a given time point (19,20). Because an abutment change affects the ISQ values, RFA data were not collected after replacing a longer or shorter abutment.

Soft tissue reactions were determined by the Holgers (21) scale, in which a Holgers grade 1 or higher was considered as soft tissue reaction, and a Holgers grade 2 or higher was considered as *adverse* soft tissue reaction. Furthermore, implant loss or explantation was recorded. Implant loss or explantation was registered with the reason for loss or explantation, and the date the loss occurred or the implant was explanted. Lastly, the extent of sound processor usage was recorded. In selective users or nonusers, the reason for selective use/nonuse was also noted.

### Statistical Analysis and Data Management

No new sample size calculations were performed; all patients from the previous investigations were asked to participate. For the original study, a power calculation was conducted on the primary outcome variable, ISQ-high (7). A correction factor was developed and validated by Ostell AB to enable comparison between ISQ values measured with different SmartPegs used throughout the 10-year follow-up. For comparisons between test and control groups, Mann-Whitney *U* tests were used for continuous variables, Mantel-Haenszel *χ*^2^ tests were used for ordered variables, and Fisher exact tests were used for dichotomous variables. Wilcoxon signed rank tests were used to analyze changes within groups for continuous variables. Implant survival was analyzed using Kaplan-Meier survival curves with log-rank tests. Implants in telephonically unreachable patients or patients without recently available information regarding implant survival were considered as “surviving” (n = 1). A significance level of 0.05 was adopted, and all tests were two-tailed. No corrections were made for multiple comparisons.

According to a predefined statistical analysis plan, an external data manager (Statistiska Konsultgruppen, Göteborg, Sweden) performed data management and statistical analysis.

### Ethical Considerations

Because of the nature of this study, this study was exempt from obtaining ethical approval by the local ethical committee. This study was performed according to the guidelines for Good Clinical Practice, ISO14155:2011, and ethical principles stated by the Declaration of Helsinki (22). All included patients provided written informed consent. The current study was registered at ClinicalTrials.gov under identifier NCT05058066.

## RESULTS

### Patients and Follow-up

Of the 72 patients from the original trials (with 42 trial 1 and 30 trial 2 patients), 51 patients attended the 10-year follow-up. Twenty-one patients did or could not attend the 10-year follow-up because they were deceased (n = 2), lost the implant (n = 6), underwent explantation of the implant (n = 1) or removal of the abutment (n = 1), underwent replacement of the abutment (n = 2), were unreachable (n = 2), or were not able or willing to visit the clinic physically (n = 7). Only one nondeceased patient (group B), who visited the clinic 8 years after implantation for the last time (with the implant in situ), was unreachable.

As described earlier, groups A and B were compared in the previous trial 1, and groups B and C were compared in the previous trial 2. Group A, the patients who received a control implant that was loaded 6 weeks after surgery, consisted of 7 patients; group B, the patients who received a test implant that was loaded 6 weeks after surgery, consisted of 23 patients; and group C, the patients who received a test implant that was loaded 3 weeks after surgery, consisted of 21 patients. The patients who underwent removal or replacement of the abutment, or could not attend the physical visits, were included in the implant survival population but excluded in the other follow-up analyses. There were no significant differences in baseline characteristics between study groups (Table 1).

**TABLE 1:** Baseline characteristics

Variable	10-yr Follow-Up Population (n = 51)				
	6-wk Loading (n = 30)			3-wk Loading (n = 21)	
	Control Implant (n = 7)	Test Implant (n = 23)	*p*		*p*
Sex, n (%)			1.00		0.53
Female	3 (42.9)	11 (47.8)		13 (61.9)	
Male	4 (57.1)	12 (52.2)		8 (38.1)	
Age (yr), mean (SD)	64.7 (16.2)	66.3 (10.0)	0.71	66.2 (11.0)	0.96

	10-yr Follow-up Population (n = 51)			Implant Survival Population (n = 72)		
Variable	Control Implant (n = 7)	Test Implant (n = 44)	*p*	Control Implant (n = 14)	Test Implant (n = 58)	*p*
Sex, n (%)			0.86			0.37
Female	3 (35.7)	24 (54.5)		5 (35.7)	31 (53.4)	
Male	4 (64.3)	20 (45.5)		9 (64.3)	27 (46.6)	
Age (yr), mean (SD)	64.7 (16.2)	66.3 (10.3)		67.8 (12.0)	65.1 (11.3)	0.34
Indication for BAHI, n (%)			0.13			0.53
Conductive	3 (42.9)	8 (34.8)		6 (42.9)	9 (32.1)	
Mixed	3 (42.9)	8 (34.8)		5 (35.7)	8 (28.6)	
SSD	0 (0.0)	7 (30.4)		2 (14.3)	10 (35.7)	
Other	1 (14.3)	0 (0.0)		1 (7.1)	1 (3.6)	

BAHI indicates bone-anchored hearing implant; SD, standard deviation; SSD, single-sided deafness.

### Implant Stability Quotient

This section will only describe ISQ-high values (further referred to as “ISQ values”) and omit ISQ-low values to avoid mentioning similar results. The mean ISQ values of group B were significantly higher than the values of group A at all measuring points. There were no significant differences between the mean ISQ values of groups B and C throughout all postoperative visits during the first 6 months after implantation. At all subsequent follow-up visits, the mean ISQ values of group B were significantly higher than those of group C, except for the 3-year follow-up visit (*p* = 0.84). The mean ISQ at 5 years was 70.8 (standard deviation [SD], ±2.9) for groups A and B together compared with 66.8 (SD, ±5.0) for group C (*p* < 0.001). The mean ISQ at 10 years was 62.0 (SD, ±6.6) for group A compared with 70.1 (SD, ±2.4) for group B (*p* = 0.002) and 66.2 (SD, ±6.0) for group C (*p* = 0.031).

**FIG. 1:**
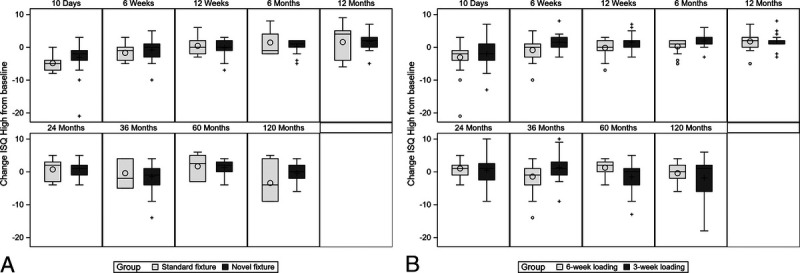
*A*, Box-and-whisker plot of the change in ISQ-high by implant type based on follow-up time compared with baseline ISQ-high. Mean (*○ and +*) and median (*horizontal line*) are defined within in the boxplot. The *box* represents the interquartile range, the *whiskers* the 95% confidence interval, and the single *plus signs* the outliers. *B*, Box-and-whisker plot of the change in ISQ-high by loading group based on follow-up time compared with baseline ISQ-high. Mean (*○ and +*) and median (*horizontal line*) are defined within in the boxplot. The *box* represents the interquartile range, the *whiskers* the 95% confidence interval, and the single *plus signs* the outliers. ISQ indicates implant stability quotient.

**FIG. 2:**
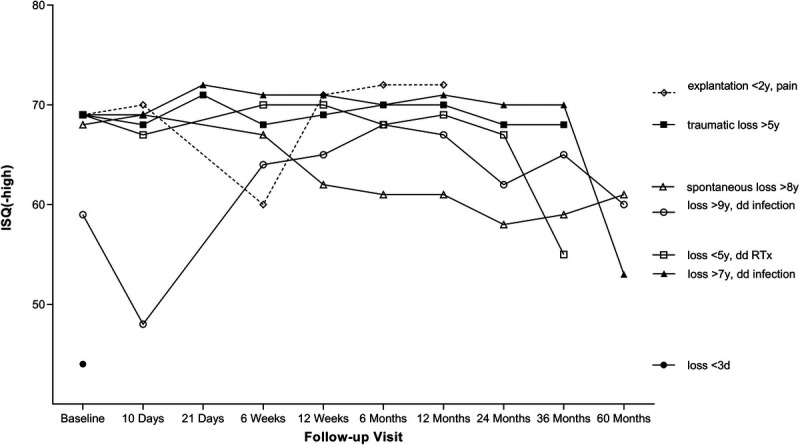
ISQ-high values per lost/explanted implant per follow-up visit. ISQ indicates implant stability quotient.

A decrease in mean ISQ was recorded for all groups between the 5-year follow-up visit and the 10-year follow-up visit. This decrease was also recorded between the 3-year follow-up and the 5-year follow-up visit for group C. The change in ISQ from the first postoperative visit to 10 years was 1.58 (SD, ±6.16; within group, *p* = 0.44) for the test implant groups (groups B and C) and 2.65 (SD, ±5.59; within group, *p* = 0.80) for the control implant (group A). No differences were noticed in the change of mean ISQ values from baseline to 10 years between groups A–B and B–C. Figure 1 shows the ISQ change from baseline to 10-year follow-up visit for both implants (Fig. 1A) and loading groups (Fig. 1B) as box-and-whisker plots. Figure 2 shows the ISQ values as a function of time/follow-up visits for the patients that lost their implant (n = 7).

### Soft Tissue Reactions

**FIG. 3:**
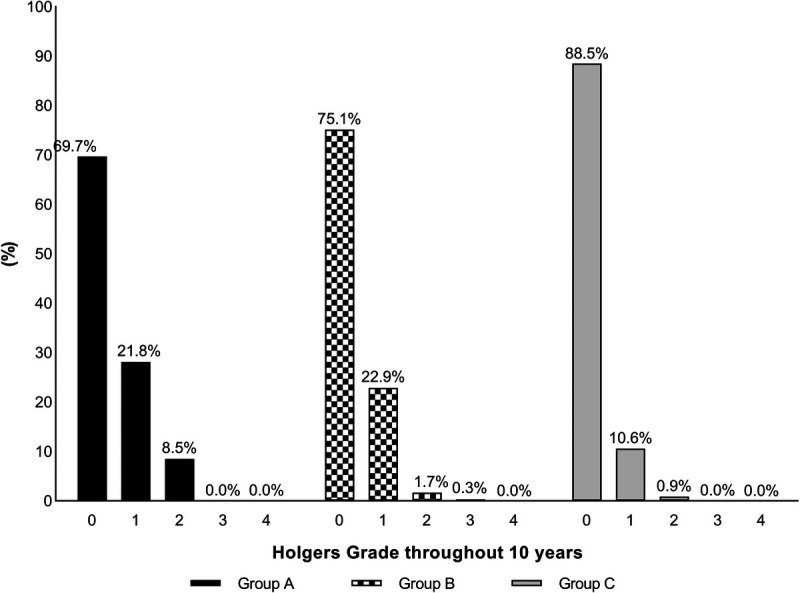
Soft tissue reactions as a percentage of visits according to Holgers classification observed for the study population (groups A, B, and C) throughout the entire 10-year follow-up. Holgers grade 0 signifies normal skin; Holgers grade 1 indicates slight redness; Holgers grade 2 refers to red and slightly moist tissue; Holgers grade 3 means the formation of tissue granulation around the abutment.

At the 10-year follow-up visit, a Holgers 0 score was observed in almost all patients. In only one patient (4.3%), a mild soft tissue reaction (Holgers 1) was observed, which occurred in a group B patient. No soft tissue reactions requiring treatment, also known as adverse soft tissue reactions (Holgers ≥2), were observed. Figure 3 provides an overview of soft tissue reactions observed throughout the 10-year follow-up.

### Implant Survival

**FIG. 4:**
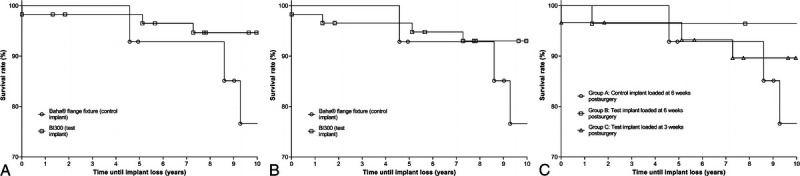
*A*, Implant survival, excluding explantations: lines represent the survival curve for both implant types (*p* = 0.050). *B*, Implant survival, including explantations: lines represent the survival curve for both implant types (*p* = 0.106). *C*, Implant survival, including explantations: lines represent the survival curve for all three study groups (*p* = 0.188).

Independent of the loading time, the test implant showed significantly better survival than the control implant at 10 years of follow-up (94.8% versus 78.6%, *p* = 0.050; Fig. 4A). With explantations included, the test still “survived” better than the control implant, but insignificantly (93.1% versus 78.6%, *p* = 0.106; Fig. 4B). During the first 5 years of the study, one implant was lost in group A. This occurred almost 5 years after implantation and was possibly related to radiotherapy at the implant site in the months before the loss. No implants were explanted. In group B, one implant was explanted because of chronic pain around the abutment, and no implants were lost. In group C, one implant was lost as well, which occurred 3 days after implantation.

Between the 5- and 10-year visits, another two implants were lost in group A. No implants were explanted. The first of these losses between the 5- and 10-year visits occurred spontaneously 8 years after implantation. After reimplantation, implant loss occurred again, despite the lack of risk factors related to implant loss in the patients' medical history. The other implant loss between the 5- and 10-year visits occurred 9 years after implantation, possibly associated with an infection at the implant site. In group B, no implants were lost or explanted. In group C, two implants were lost. The first loss occurred 5 years after implantation, after the 5-year visit, and was caused by trauma. The second loss, possibly related to an infection, occurred around 7 years after implantation.

Excluding explantations, the implant survival rate was 78.6% for the control implant loaded at 6 weeks (group A), 100% for the test implant loaded at 6 weeks (group B), and 90.0% for the test implant loaded at 3 weeks (group C). The survival rates, including explantations, were 78.6, 96.4, and 90.0%, respectively.

### Sound Processor Usage

In total, 53 patients were asked to what extent they used their sound processor. Forty-eight patients made use of their sound processor, of which 38 patients daily and 36 patients the entire day (≥16 hours). Eleven patients reported selective use of their sound processor, for example, during school or work, watching television, or only during periods of otitis (once/twice a year). Five patients were nonusers. Reasons for nonuse varied widely: dissatisfaction with the device's (mechanical) sound, alternate usage of a conventional Contralateral Routing Of Signals device, or the alternate use of a headset since working from home. Mean sound processor use at 10 years was 5.6 (SD, ±2.55) days a week and 12.1 (SD, ±6.33) hours a day. Two patients even reported using their sound processor at night: one patient during the weeks her husband was hospitalized and one bilaterally fitted patient alternated between the two implants every other night.

## DISCUSSION

### Key Results

This study compared the implant stability, survival, and soft tissue reactions of a wide-diameter implant-abutment combination (test) with a previous-generation implant and abutment (control) 10 years after implantation. Furthermore, the safety of loading the test implant 3 weeks after surgery was assessed. The implant survival of the test implant proved to be significantly better than the control implant at the 10 years of follow-up, without explantations. Survival rates of both loading groups remained high without significant differences between both loading groups. In addition, the test implant showed superiority in terms of higher mean ISQ values during the entire 10-year follow-up. Soft tissue reactions were scarcely found in both implant and loading groups, with only one Holgers 1 in group C and no Holgers 2 or higher at the 10-year follow-up visit in any group.

### Strengths and Limitations

This study is the first prospective trial comparing the clinical outcomes between a wide-diameter implant and a previous-generation small-diameter implant at long-term (10-year) follow-up. In addition, it is the first prospective trial that compares the 10-year results between loading of a BAHI 3 weeks after surgery with those 6 weeks after surgery. The original controlled and partially randomized trials with multiple participating centers provided strong evidence for high implant stability, implant survival, and good soft tissue outcomes until 5 years of follow-up. With the additional long-term follow-up in a prospective manner and original setup, we confirmed these excellent outcomes for the test implant, even when it was loaded 3 weeks after surgery.

Because of its combined nature, there are limitations to this study that are worth mentioning. One limitation is that not all follow-up visits were synchronous with each other. This unsynchronized visit concerned only a slight difference in time (1 week) and the absence of one follow-up visit (8 weeks). Furthermore, as already described in the 5-year study (8), another limitation is the loss of follow-up of some patients compared with the original, powered study sample. Twenty-one patients could not be included in the 10-year analysis of implant stability and soft tissue reactions. Consequently, a selection bias for the last two follow-up visits cannot be excluded, even more because the current visits were a distinct investigation for which patients had to give separate informed consent. All 72 patients of the original studies were included in the implant survival analysis; however, survival information was based on patient files and/or information collected in the initial investigations for the patients who could not be contacted.

### Interpretation and Comparison With Other Studies

Most investigations concerning the wide-diameter implant are retrospective studies without a control group or small pilots, all with a relatively short-term follow-up (12–15,23–26). To obtain more evidence on clinically important outcomes like implant survival, it would be highly desirable to have long-term follow-up studies. With its 10 years of postsurgical follow-up, the current investigation desires to overcome this gap from retrospective, short follow-up studies.

Implant stability as measured by RFA was initially chosen as the primary outcome measure. To date, RFA is still the only objective technique available to measure implant stability. However, as Nelissen et al. (19) and den Besten et al. (8) pointed out, it should be interpreted with caution. The ISQ is influenced by implant and abutment design, surgery, SmartPeg type, and the person that measures. This subjectiveness tends to lead to the idea that an isolated ISQ value provides incomplete information. Hence, the clinical use of RFA should focus on ISQ trends because they could indicate implant stability failure. Therefore, it was recommended to use RFA only in longitudinal studies (19).

During this study, ISQ values were measured consequently for 10 years. Indeed, the patients who lost their implant during follow-up because of decreased bone quality (e.g., due to radiotherapy to implant site) or infection showed a downward trend in ISQ values. Remarkably, the ISQ values seemed to disperse increasingly over time. This remarkable dispersion appeared particularly in our groups A and C, which matches their higher implant loss rate but makes us question the value of RFA, besides its objectiveness, even more. This study again emphasizes that ISQ changes must be considered not only over the “longer run” but also together with clinical information about the patient. When a patient reports (chronic) pain at the implant site and a periprosthetic infection is plausible, ISQ could be of added value in predicting implant loss. Bearing this in mind, implant loss remains the most evident, and therefore most relevant, outcome measure. This study underlines that implant loss can still occur after years but with shallow risk. This risk is significantly reduced after implantation with a wide-diameter implant. Furthermore, and of clinical importance, implant loss risk did not increase in wide implants loaded 3 weeks after surgery. This result confirms that loading the implant with the sound processor 3 weeks after surgery is safe.

Lastly, the question arises whether it is safe to reduce loading times even further. In the light of patient-centered care, it is essential to take the patients' preferences and perspectives regarding postsurgical loading time into account. Caspers et al. (27) found that patients do not always necessarily prefer earlier loading: preoperatively, 70% of all patients preferred sound processor loading within 1 week after surgery, but interestingly, 3 weeks after surgery, significantly more patients preferred loading at a later moment.

## CONCLUSIONS

The tested wide-diameter implant with abutment showed significantly better implant survival during the 10 years of follow-up compared with the previous-generation implant with abutment in adult patients with normal bone quality. Although BAHI surgery was already a safe procedure, a persistent reduction in soft tissue reactions was noticed 10 years after implantation for both implants. At the most extended follow-up reported to date (10 years) in a prospective controlled study, it can be concluded that the BIA300 has good long-term implant stability, survival, and tolerability, even after loading it 3 weeks after surgery.
